# Supplementary material for: Lipidation-independent vacuolar functions of Atg8 rely on its noncanonical interaction with a vacuole membrane protein
Source: eLife. 2018 Nov 19;7:e41237. doi: 10.7554/eLife.41237 (PMC6279349; doi:10.7554/eLife.41237)
Supplement: Supplementary file 2. [file elife-41237-supp2.docx]

**Table S1. Data collection and refinement statistics.**

|  | SpAtg8-SpHfl1 | ScAtg8-ScHfl1 |
| --- | --- | --- |
| *Data Statistics* |  |  |
| Beamline | SPring-8 BL32XU | KEK BL-5A |
| Wavelength (Å) | 1.0000 | 1.0000 |
| Space group | *P*2_1_2_1_2 | *P*2_1_ |
| Cell parameters *a* (Å) | 33.80 | 90.47 |
| Cell parameters *b* (Å) | 108.87 | 61.65 |
| Cell parameters *c* (Å) | 35.12 | 100.11 |
| Cell parameters *β* (˚) |  | 96.37 |
| Resolution range (Å) | 35.12–2.20 | 45.73-2.45 |
| Outer range (Å) | 2.33-2.20 | 2.49-2.45 |
| Observed reflections | 45,171 | 134,956 |
| Unique reflections | 7,091 | 40,557 |
| Completeness (%) | 99.8 (98.9) | 99.6 (99.5) |
| *R*_merge_ (I) | 0.122 (1.331) | 0.112 (0.647) |
| <I/σ(I)> | 8.1 (1.1) | 6.5 (1.1) |
| *Refinement statistics* |  |  |
| Resolution range (Å) | 35.12-2.20 | 45.73-2.45 |
| No. of protein atoms | 1,085 | 7,664 |
| No. of water | 4 | 195 |
| *R* / *R*_free_ | 0.211/0.259 | 0.193/0.244 |
| Rmsd bond length (Å) | 0.004 | 0.004 |
| Rmsd bond angle (º) | 0.73 | 0.79 |

Values in parentheses refer to the outer shell.

Ramachandran plot calculated by Rampage (Lovell et al., 2003).

SpHfl1: favoured 96.9%, allowed 3.1%, outlier 0.0%.

ScHfl1: favoured 97.4%, allowed 2.6%, outlier 0.0%.

Table S2.

Fission yeast strains used in this study.

| Strain | Genotype | |
| --- | --- | --- |
| LD328 | *h+* | *leu1-32 his3-D1* |
| DY6142 | *h-* | *leu1-32 his3-D1* |
| DY43906 | *h?* | *leu1-32 his3-D1? Patg8-mYFP-atg8::kanMX hfl1-mCherry::natMX* |
| DY43907 | *h-* | *leu1-32 Patg8-mYFP-atg8::kanMX* |
| DY43908 | *h+* | *leu1-32 his3-D1 hfl1-mCherry::natMX* |
| DY43909 | *h?* | *leu1-32 his3-D1 hfl1-GFP::hphMX zhf1-mCherry::natMX* |
| DY43910 | *h?* | *leu1-32 his3-D1 Patg8-mYFP-atg8::kanMX zhf1-mCherry::natMX* |
| DY43911 | *h?* | *leu1-32 his3-D1 Patg8-mYFP-atg8::kanMX zhf1-mCherry::natMX hfl1Δ::kanMX* |
| DY43912 | *h-* | *leu1-32 his3-D1 hfl1Δ::kanMX Patg8-mYFP-atg8::kanMX leu1-32::Pnmt1-hfl1-mCherry(leu1+)* |
| DY43913 | *h?* | *leu1-32 his3-D1 hmt1-mCherry::kanMX cpy1-Venus::hphMX* |
| DY43914 | *h-* | *leu1-32 his3-D1 hmt1-mCherry::kanMX cpy1-Venus::hphMX hfl1Δ::hphMX* |
| DY43915 | *h+* | *leu1-32 his3-D1 hmt1-mCherry::kanMX cpy1-Venus::natMX atg8Δ::kanMX* |
| DY43916 | *h-* | *leu1-32 his3-D1 atg8Δ::kanMX* |
| DY43917 | *h+* | *leu1-32 his3-D1 hfl1Δ::kanMX* |
| DY43918 | *h?* | *leu1-32 his3-D1 atg8Δ::kanMX hfl1Δ::hphMX* |
| DY43919 | *h+* | *leu1-32 his3-D1 atg8-G116A::kanMX* |
| DY43920 | *h-* | *leu1-32 his3-D1 atg7Δ::kanMX* |
| DY43921 | *h+* | *leu1-32 his3-D1 atg3Δ::kanMX* |
| DY26196 | *h-* | *leu1-32 CFP-atg8::leu1+* |
| DY43922 | *h+* | *leu1-32 CFP-atg8::leu1+ hfl1Δ::hphMX* |
| DY11899 | *h-* | *leu1-32 CFP-atg8::leu1+ atg3Δ::kanMX* |
| DY4003 | *h?* | *leu1-32 CFP-atg8::leu1+ atg4Δ::kanMX* |
| DY4021 | *h?* | *leu1-32 CFP-atg8::leu1+ atg5Δ::kanMX* |
| DY43923 | *h-* | *leu1-32 CFP-atg8::leu1+ atg16Δ::hphMX* |
| DY43924 | *h-* | *leu1-32 his3-D1 cpy1-mCherry::natMX* |
| DY43925 | *h?* | *leu1-32 his3-D1 cpy1-mCherry::natMX atg1Δ::kanMX* |
| DY43926 | *h?* | *leu1-32 his3-D1 cpy1-mCherry::natMX atg2Δ::kanMX* |
| DY43927 | *h?* | *leu1-32 his3-D1 cpy1-mCherry::natMX atg3Δ::kanMX* |
| DY43928 | *h?* | *leu1-32 his3-D1 cpy1-mCherry::natMX atg4Δ::kanMX* |
| DY43929 | *h?* | *leu1-32 his3-D1 cpy1-mCherry::natMX atg5Δ::kanMX* |
| DY43930 | *h?* | *leu1-32 his3-D1 cpy1-mCherry::natMX atg6Δ::kanMX* |
| DY43931 | *h?* | *leu1-32 his3-D1 cpy1-mCherry::natMX atg7Δ::kanMX* |
| DY43932 | *h?* | *leu1-32 his3-D1 cpy1-mCherry::natMX atg8-G116A::kanMX* |
| DY43933 | *h+* | *his3-D1 ura4-D18 atg4Δ::kanMX leu1-32::Pnmt1-atg8-mCherry(leu1+)* |
| DY43934 | *h+* | *his3-D1 ura4-D18 atg4Δ::kanMX leu1-32::Pnmt1-atg8-mCherry(leu1+) ars1::Pnmt1-hfl1-GFP(ura4+)* |
| DY43935 | *h+* | *his3-D1 ura4-D18 atg4Δ::kanMX leu1-32::Pnmt1-atg8-mCherry(leu1+) ars1::Pnmt1-hfl1-Δ(386-409)-GFP(ura4+)* |
| DY43936 | *h+* | *his3-D1 ura4-D18 atg4Δ::kanMX leu1-32::Pnmt1-atg8-mCherry(leu1+) ars1::Pnmt1-hfl1-(1-269)-GFP(ura4+)* |
| DY43937 | *h+* | *his3-D1 ura4-D18 atg4Δ::kanMX leu1-32::Pnmt1-atg8-mCherry(leu1+) ars1::Pnmt1-hfl1-(1-385)-GFP(ura4+)* |
| DY43938 | *h+* | *his3-D1 ura4-D18 atg4Δ::kanMX leu1-32::Pnmt1-atg8-mCherry(leu1+) ars1::Pnmt1-hfl1-(1-410)-GFP(ura4+)* |
| DY43939 | *h+* | *his3-D1 ura4-D18 atg4Δ::kanMX leu1-32::Pnmt1-atg8-mCherry(leu1+) ars1::Pnmt1-hfl1-(270-426)-GFP(ura4+)* |
| DY43940 | *h-* | *his3-D1 hfl1Δ::kanMX* *Patg8-mYFP-atg8::kanMX leu1-32::Pnmt41-hfl1-mCherry(leu1+)* |
| DY43941 | *h-* | *his3-D1 hfl1Δ::kanMX* *Patg8-mYFP-atg8::kanMX leu1-32::Pnmt41-mCherry(leu1+)* |
| DY43942 | *h-* | *his3-D1 hfl1Δ::kanMX* *Patg8-mYFP-atg8::kanMX leu1-32::Pnmt41-hfl1-(1-269)-mCherry(leu1+)* |
| DY43943 | *h-* | *his3-D1 hfl1Δ::kanMX* *Patg8-mYFP-atg8::kanMX leu1-32::Pnmt41-hfl1-(1-317)-mCherry(leu1+)* |
| DY43944 | *h-* | *his3-D1 hfl1Δ::kanMX* *Patg8-mYFP-atg8::kanMX leu1-32::Pnmt41-hfl1-(1-361)-mCherry(leu1+)* |
| DY43945 | *h-* | *his3-D1 hfl1Δ::kanMX* *Patg8-mYFP-atg8::kanMX leu1-32::Pnmt41-hfl1-(1-385)-mCherry(leu1+)* |
| DY43946 | *h-* | *his3-D1 hfl1Δ::kanMX* *Patg8-mYFP-atg8::kanMX leu1-32::Pnmt41-hfl1-(1-410)-mCherry(leu1+)* |
| DY43947 | *h-* | *his3-D1 hfl1Δ::kanMX* *Patg8-mYFP-atg8::kanMX leu1-32::Pnmt41-hfl1-Δ(386-409)-mCherry(leu1+)* |
| DY43948 | *h+* | *his3-D1 hfl1Δ::kanMX* *cpy1-Venus::hphMX* *leu1-32::Pnmt41-hfl1-mCherry(leu1+)* |
| DY43949 | *h+* | *his3-D1 hfl1Δ::kanMX* *cpy1-Venus::hphMX* *leu1-32::Pnmt41-mCherry(leu1+)* |
| DY43950 | *h+* | *his3-D1 hfl1Δ::kanMX* *cpy1-Venus::hphMX* *leu1-32::Pnmt41-hfl1-(1-269)-mCherry(leu1+)* |
| DY43951 | *h+* | *his3-D1 hfl1Δ::kanMX* *cpy1-Venus::hphMX* *leu1-32::Pnmt41-hfl1-Δ(386-409)-mCherry(leu1+)* |
| DY43952 | *h+* | *his3-D1 hfl1Δ::kanMX* *cpy1-Venus::hphMX* *leu1-32::Pnmt41-hfl1-(1-317)-mCherry(leu1+)* |
| DY43953 | *h+* | *his3-D1 hfl1Δ::kanMX* *cpy1-Venus::hphMX* *leu1-32::Pnmt41-hfl1-(1-361)-mCherry(leu1+)* |
| DY43954 | *h+* | *his3-D1 hfl1Δ::kanMX* *cpy1-Venus::hphMX* *leu1-32::Pnmt41-hfl1-(1-385)-mCherry(leu1+)* |
| DY43955 | *h+* | *his3-D1 hfl1Δ::kanMX* *cpy1-Venus::hphMX* *leu1-32::Pnmt41-hfl1-(1-410)-mCherry(leu1+)* |
| DY43956 | *h-* | *his3-D1 hfl1Δ::kanMX* *Patg8-mYFP-atg8::kanMX leu1-32::Pnmt41-hfl1-L397A-mCherry(leu1+)* |
| DY43957 | *h-* | *his3-D1 hfl1Δ::kanMX* *Patg8-mYFP-atg8::kanMX leu1-32::Pnmt41-hfl1-Y398A-mCherry(leu1+)* |
| DY43958 | *h-* | *his3-D1 hfl1Δ::kanMX* *Patg8-mYFP-atg8::kanMX leu1-32::Pnmt41-hfl1-E393A-mCherry(leu1+)* |
| DY43959 | *h-* | *his3-D1 hfl1Δ::kanMX* *Patg8-mYFP-atg8::kanMX leu1-32::Pnmt41-hfl1-E395A-mCherry(leu1+)* |
| DY43960 | *h-* | *his3-D1 hfl1Δ::kanMX* *Patg8-mYFP-atg8::kanMX leu1-32::Pnmt41-hfl1-F388A-mCherry(leu1+)* |
| DY43961 | *h+* | *his3-D1 hfl1Δ::kanMX* *Patg8-mYFP-atg8::kanMX leu1-32::Pnmt41-hfl1-Y411A-mCherry(leu1+)* |
| DY43962 | *h+* | *his3-D1 hfl1Δ::kanMX* *Patg8-mYFP-atg8::kanMX leu1-32::Pnmt41-hfl1-L386A-mCherry(leu1+)* |
| DY43963 | *h+* | *his3-D1 hfl1Δ::kanMX* *cpy1-Venus::hphMX* *leu1-32::Pnmt41-hfl1-Y398A-mCherry(leu1+)* |
| DY43964 | *h+* | *his3-D1 hfl1Δ::kanMX* *cpy1-Venus::hphMX* *leu1-32::Pnmt41-hfl1-E395A-mCherry(leu1+)* |
| DY43965 | *h+* | *his3-D1 hfl1Δ::kanMX* *cpy1-Venus::hphMX* *leu1-32::Pnmt41-hfl1-E393A-mCherry(leu1+)* |
| DY43966 | *h+* | *his3-D1 hfl1Δ::kanMX* *cpy1-Venus::hphMX* *leu1-32::Pnmt41-hfl1-L397A-mCherry(leu1+)* |
| DY43967 | *h+* | *his3-D1 hfl1Δ::kanMX* *cpy1-Venus::hphMX* *leu1-32::Pnmt41-hfl1-Y411A-mCherry(leu1+)* |
| DY43968 | *h+* | *his3-D1 hfl1Δ::kanMX* *cpy1-Venus::hphMX* *leu1-32::Pnmt41-hfl1-L386A-mCherry(leu1+)* |
| DY43969 | *h+* | *his3-D1 hfl1Δ::kanMX* *cpy1-Venus::hphMX* *leu1-32::Pnmt41-hfl1-F388A-mCherry(leu1+)* |
| DY43980 | *h+* | *his3-D1 cpy1-Venus::hphMX hfl1Δ::kanMX leu1-32::Pnmt41-hfl1-mCherry(leu+)* |
| DY43981 | *h+* | *his3-D1 cpy1-Venus::hphMX hfl1Δ::kanMX leu1-32::Pnmt41-hfl1-mCherry-atg8(leu+)* |
| DY43982 | *h+* | *his3-D1 cpy1-Venus::hphMX hfl1Δ::kanMX leu1-32::Pnmt41-hfl1-Y398A-mCherry(leu+)* |
| DY43983 | *h90* | *his3-D1 cpy1-Venus::hphMX hfl1Δ::kanMX leu1-32::Pnmt41-hfl1-Y398A-mCherry-atg8(leu+)* |
| DY43984 | *h+* | *his3-D1 cpy1-Venus::hphMX hfl1Δ::kanMX leu1-32::Pnmt41-mCherry(leu+)* |
| DY43990 | *h+* | *his3-D1 cpy1-Venus::hphMX hfl1Δ::kanMX leu1-32::Pnmt41-mCherry-atg8(leu+)* |
| DY43991 | *h+* | *his3-D1 cpy1-Venus::hphMX hfl1Δ::kanMX leu1-32::Pnmt41-zhf1-mCherry (leu+)* |
| DY43992 | *h+* | *his3-D1 cpy1-Venus::hphMX hfl1Δ::kanMX leu1-32::Pnmt41- zhf1-mCherry -atg8(leu+)* |
| DY43993 | *h+* | *his3-D1 cpy1-Venus::hphMX hfl1Δ::kanMX leu1-32::Pnmt41-hmt1-mCherry (leu+)* |
| DY43994 | *h+* | *his3-D1 cpy1-Venus::hphMX hfl1Δ::kanMX leu1-32::Pnmt41- hmt1-mCherry -atg8(leu+)* |
| DY43995 | *h+* | *leu1-32 his3-D1 ura4-D18 atg4Δ::kanMX ars1::atg8-YFH(ura4+)* |

**Table S3.**

Budding yeast strains used in this study.

| Strain | Genotype | |
| --- | --- | --- |
| DY30422 | **a** | *his3D1 lys2D0 ura3D0 Schfl1Δ::natMX leu2D0::pNH605-pTEF1-ScHFL1-GFP(CgLEU2)* |
| DY30428 | **a** | *his3D1 met15D0 ura3D0 atg4Δ::kanMX ScATG8-mCherry::natMX*  *Schfl1Δ::hphMX leu2D0::pNH605-pTEF1-ScHFL1-GFP(CgLEU2)* |
| DY27780 | **a** | *his3D1 leu2D0 met15D0 ura3D0 Schfl1Δ::hphMX atg4Δ::kanMX ScATG8-mCherry::natMX* |
| DY28168 | **a** | *his3D1 leu2D0 met15D0 ura3D0 atg4Δ::kanMX ScATG8-mCherry::natMX ScHFL1-GFP(S65T)::hphMX* |
| DY27670 (CWY7129) | **a** | *his3D1 leu2D0 lys2D0 ura3D0 VPH1-mCherry::LEU* |
| DY27674 (CWY7220) | **a** | *his3D1 leu2D0 lys2D0 ura3D0 VPH1-mCherry::LEU*  *Scatg8Δ::kanMX* |
| DY27919 | **a** | *his3D1 leu2D0 lys2D0 ura3D0 VPH1-mCherry::LEU*  *Schfl1Δ::natMX* |
| DY27671 (CWY7204) | **a** | *his3D1 leu2D0 lys2D0 ura3D0 VPH1-mCherry::LEU atg1Δ::hphMX* |
| DY27673 (CWY7216) | **a** | *his3D1 leu2D0 lys2D0 ura3D0 VPH1-mCherry::LEU atg7Δ::hphMX* |
| DY43889 | **a** | *his3D1 leu2D0 lys2D0 VPH1-mCherry::LEU ura3D0::GFP-ScATG8(KlURA3)* |
| DY43890 | **a** | *his3D1 leu2D0 lys2D0 VPH1-mCherry::LEU Scatg7Δ::hphMX ura3D0::GFP-ScATG8(KlURA3)* |
| DY43891 | **a** | *his3D1 leu2D0 lys2D0 VPH1-mCherry::LEU Schfl1Δ::natMX ura3D0::GFP-ScATG8(KlURA3)* |
| DY32268 | **a** | *his3D1 met15D0 ura3D0 atg4Δ::kanMX hfl1Δ::hphMX ScATG8-mCherry::natMX leu2D0::pNH605-pTEF1-GFP(CgLEU2)* |
| DY32270 | **a** | *his3D1 met15D0 ura3D0 atg4Δ::kanMX hfl1Δ::hphMX ScATG8-mCherry::natMX leu2D0::pNH605-pTEF1-ScHFL1-W371A-GFP(CgLEU2)* |
| DY32272 | **a** | *his3D1 met15D0 ura3D0 atg4Δ::kanMX hfl1Δ::hphMX ScATG8-mCherry::natMX leu2D0::pNH605-pTEF1-ScHFL1-I375A-GFP(CgLEU2)* |
| DY43892 | **a** | *his3D1 met15D0 ura3D0 atg4Δ::kanMX hfl1Δ::hphMX ScATG8-mCherry::natMX leu2D0::pNH605-pTEF1-ScHFL1-D384A-GFP(CgLEU2)* |
| DY43893 | **a** | *his3D1 met15D0 ura3D0 atg4Δ::kanMX hfl1Δ::hphMX ScATG8-mCherry::natMX leu2D0::pNH605-pTEF1-ScHFL1-Y387A-GFP(CgLEU2)* |
| DY32274 | **a** | *his3D1 met15D0 ura3D0 atg4Δ::kanMX hfl1Δ::hphMX ScATG8-mCherry::natMX leu2D0::pNH605-pTEF1-ScHFL1-W371AI375A-GFP(CgLEU2)* |
| DY43894 | **a** | *his3D1 met15D0 ura3D0 atg4Δ::kanMX hfl1Δ::hphMX ScATG8-mCherry::natMX leu2D0::pNH605-pTEF1-ScHFL1-W371AI375AD384AY387A-GFP(CgLEU2)* |
| DY33416 | **a** | *his3D1 lys2D0 ura3D0 VPH1-mCherry::kanMX Schfl1Δ::hphMX leu2D0::pNH605-pTEF1-GFP(**CgLEU2)* |
| DY33085 | **a** | *his3D1 lys2D0 ura3D0 VPH1-mCherry::kanMX Schfl1Δ::hphMX leu2D0::pNH605-pTEF1-ScHFL1-GFP(CgLEU2)* |
| DY33408 | **a** | *his3D1 lys2D0 ura3D0 VPH1-mCherry::kanMX Schfl1Δ::hphMX leu2D0::pNH605-pTEF1-ScHFL1-W371A-GFP(CgLEU2)* |
| DY33412 | **a** | *his3D1 lys2D0 ura3D0 VPH1-mCherry::kanMX Schfl1Δ::hphMX leu2D0::pNH605-pTEF1-ScHFL1-I375A-GFP(CgLEU2)* |
| DY43895 | **a** | *his3D1 lys2D0 ura3D0 VPH1-mCherry::kanMX Schfl1Δ::hphMX leu2D0::pNH605-pTEF1-ScHFL1-D384A-GFP(CgLEU2)* |
| DY43896 | **a** | *his3D1 lys2D0 ura3D0 VPH1-mCherry::kanMX Schfl1Δ::hphMX leu2D0::pNH605-pTEF1-ScHFL1-Y387A-GFP(CgLEU2)* |
| DY33414 | **a** | *his3D1 lys2D0 ura3D0 VPH1-mCherry::kanMX Schfl1Δ::hphMX leu2D0::pNH605-pTEF1-ScHFL1-W371AI375A-GFP(CgLEU2)* |
| DY36443 | **a** | *his3D1 lys2D0 ura3D0 VPH1-mCherry::kanMX Schfl1Δ::hphMX leu2D0::pNH605-pTEF1-ScHFL1-W371AI375AD384AY387A-GFP(CgLEU2)* |
| DY36886 | **a** | *his3D1 lys2D0 ura3D0 VPH1-mCherry::kanMX Schfl1Δ::hphMX leu2D0::pNH605-pTEF1-ScHFL1-W371AI375AD384AY387A-GFP-ScATG8(CgLEU2)* |
| DY43897 | **a** | *his3D1 lys2D0 ura3D0 VPH1-mCherry::kanMX Schfl1Δ::hphMX leu2D0::pNH605-pURA3-ScHFL1- GFP(CgLEU2)* |
| DY43898 | **a** | *his3D1 lys2D0 ura3D0 VPH1-mCherry::kanMX Schfl1Δ::hphMX leu2D0::pNH605-pURA3-ScHFL1- W371A-GFP(CgLEU2)* |
| DY43899 | **a** | *his3D1 lys2D0 ura3D0 VPH1-mCherry::kanMX Schfl1Δ::hphMX leu2D0::pNH605-pURA3-ScHFL1- I375A-GFP(CgLEU2)* |
| DY43900 | **a** | *his3D1 lys2D0 ura3D0 VPH1-mCherry::kanMX Schfl1Δ::hphMX leu2D0::pNH605-pURA3-ScHFL1- W371AI375A-GFP(CgLEU2)* |
| DY43901 | **a** | *his3D1 lys2D0 ura3D0 VPH1-mCherry::kanMX Schfl1Δ::hphMX leu2D0::pNH605-pURA3-ScHFL1- D384A-GFP(CgLEU2)* |
| DY43902 | **a** | *his3D1 lys2D0 ura3D0 VPH1-mCherry::kanMX Schfl1Δ::hphMX leu2D0::pNH605-pURA3-ScHFL1- Y387A-GFP(CgLEU2)* |
| DY43903 | **a** | *his3D1 lys2D0 ura3D0 VPH1-mCherry::kanMX Schfl1Δ::hphMX leu2D0::pNH605-pURA3-ScHFL1- W371AI375AD384AY387A-GFP(CgLEU2)* |
| DY43904 | **a** | *his3D1 lys2D0 ura3D0 VPH1-mCherry::kanMX Schfl1Δ::hphMX leu2D0::pNH605-pURA3-GFP(CgLEU2)* |

**Table S4.**

Plasmids used in this study.

| Plasmid | Descriptive name | Description |
| --- | --- | --- |
| pDB3823 | pNH605-pTEF1-ScHFL1-GFP | pNH605 plasmid expressing ScHFL1-GFP from pTEF1 promoter |
| pDB4513 | pDUAL-Pnmt1-Hfl1-mCherry | pDUAL plasmid expressing Hfl1-mCherry from Pnmt1 promoter |
| pDB4514 | Bs-uup-uUG72-p1k-GFP-Atg8-Ura | Plasmid from Dr. Zhi-Ping Xie’s lab, expressing GFP-ScATG8 |
| pDB4515 | pDUAL-Pnmt1-Atg8-mCherry | pDUAL plasmid expressing Atg8-mCherry from Pnmt1 promoter |
| pDB4516 | pDUAL- Pnmt1-Hfl1-Δ(386-409)-GFP | pDUAL plasmid expressing Hfl1-Δ(386-409)-GFP from Pnmt1 promoter |
| pDB4517 | pDUAL- Pnmt1-Hfl1-(1-269)-GFP | pDUAL plasmid expressing Hfl1-(1-269)-GFP from Pnmt1 promoter |
| pDB4518 | pDUAL- Pnmt1-Hfl1-(1-385)-GFP | pDUAL plasmid expressing Hfl1-(1-385)-GFP from Pnmt1 promoter |
| pDB4519 | pDUAL- Pnmt1-Hfl1-(1-410)-GFP | pDUAL plasmid expressing Hfl1-(1-410)-GFP from Pnmt1 promoter |
| pDB4520 | pDUAL- Pnmt1-Hfl1-(270-426)-GFP | pDUAL plasmid expressing Hfl1-(270-426)-GFP from Pnmt1 promoter |
| pDB2159 | petDuet-his_6_-GST-Pmt3 | petDuet-his_6_-GST plasmid expressing Pmt3 |
| pDB3159 | petDuet-his_6_-GST-Yng2-PHD | petDuet-his_6_-GST plasmid expressing Yng2-PHD domain |
| pDB4521 | petDuet-his_6_-GST-ScATG8 | petDuet-his_6_-GST plasmid expressing ScATG8 |
| pDB4522 | petDuet-his_6_-GST-SpAtg8 | petDuet-his_6_-GST plasmid expressing SpAtg8 |
| pDB4523 | pDUAL-P41nmt1-Hfl1-mCherry | pDUAL plasmid expressing Hfl1-mCherry from P41nmt1 promoter |
| pDB4524 | pDUAL-P41nmt1-mCherry | pDUAL plasmid expressing mCherry from P41nmt1 promoter |
| pDB4525 | pDUAL-P41nmt1-Hfl1-(1-269)-mCherry | pDUAL plasmid expressing Hfl1-(1-269)-mCherry from P41nmt1 promoter |
| pDB4526 | pDUAL-P41nmt1-Hfl1-(1-317)-mCherry | pDUAL plasmid expressing Hfl1-(1-317)-mCherry from P41nmt1 promoter |
| pDB4527 | pDUAL-P41nmt1-Hfl1-(1-361)-mCherry | pDUAL plasmid expressing Hfl1-(1-361)-mCherry from P41nmt1 promoter |
| pDB4528 | pDUAL-P41nmt1-Hfl1-(1-385)-mCherry | pDUAL plasmid expressing Hfl1-(1-385)-mCherry from P41nmt1 promoter |
| pDB4529 | pDUAL-P41nmt1-Hfl1-(1-410)-mCherry | pDUAL plasmid expressing Hfl1-(1-410)-mCherry from P41nmt1 promoter |
| pDB4530 | pDUAL- P41nmt1-Hfl1-Δ(386-409)-mCherry | pDUAL plasmid expressing Hfl1-Δ(386-409)-mCherry from P41nmt1 promoter |
| pDB4531 | pDUAL-P41nmt1-Hfl1-L386A-mCherry | pDUAL plasmid expressing Hfl1-L386A-mCherry from P41nmt1 promoter |
| pDB4532 | pDUAL-P41nmt1-Hfl1-L397A-mCherry | pDUAL plasmid expressing Hfl1-L397A-mCherry from P41nmt1 promoter |
| pDB4533 | pDUAL-P41nmt1-Hfl1-Y398A-mCherry | pDUAL plasmid expressing Hfl1-Y398A-mCherry from P41nmt1 promoter |
| pDB4534 | pDUAL-P41nmt1-Hfl1-E393A-mCherry | pDUAL plasmid expressing Hfl1-E393A-mCherry from P41nmt1 promoter |
| pDB4535 | pDUAL-P41nmt1-Hfl1-E395A-mCherry | pDUAL plasmid expressing Hfl1-E395A-mCherry from P41nmt1 promoter |
| pDB4536 | pDUAL-P41nmt1-Hfl1-F388A-mCherry | pDUAL plasmid expressing Hfl1-F388A-mCherry from P41nmt1 promoter |
| pDB4537 | pDUAL-P41nmt1-Hfl1-Y411A-mCherry | pDUAL plasmid expressing Hfl1-Y411A-mCherry from P41nmt1 promoter |
| pDB4061 | pNH605-pTEF1-GFP | pNH605 plasmid expressing GFP from pTEF1 promoter |
| pDB4058 | pNH605-pTEF1-ScHFL1-W371A-GFP | pNH605plasmid expressing ScHFL1-W371A-GFP from pTEF1 promoter |
| pDB4059 | pNH605-pTEF1-ScHFL1-I375A-GFP | pNH605 plasmid expressing ScHFL1-I375A-GFP from pTEF1 promoter |
| pDB4538 | pNH605-pTEF1-ScHFL1-D384A-GFP | pNH605 plasmid expressing ScHFL1-D384A-GFP from pTEF1 promoter |
| pDB4539 | pNH605-pTEF1-ScHFL1-Y387A-GFP | pNH605 plasmid expressing ScHFL1-Y387A-GFP from pTEF1 promoter |
| pDB4060 | pNH605-pTEF1-ScHFL1-W371AI375A-GFP | pNH605 plasmid expressing ScHFL1-W371AI375A-GFP from pTEF1 promoter |
| pDB4540 | pNH605-pTEF1-ScHFL1-W371AI375AD384AY387A-GFP | pNH605 plasmid expressing ScHFL1-W371AI375AD384AY387A-GFP from pTEF1 promoter |
| pDB4541 | pNH605-pTEF1-ScHFL1-W371AI375AD384AY387A-GFP-ScATG8 | pNH605 plasmid expressing ScHFL1-W371AI375AD384AY387A-GFP-ScATG8 from pTEF1 promoter |
| pDB4542 | pNH605-pURA3-ScHFL1-GFP | pNH605 plasmid expressing ScHFL1-GFP from pURA3 promoter |
| pDB4543 | pNH605-pURA3-ScHFL1-W371A-GFP | pNH605 plasmid expressing ScHFL1-W371A-GFP from pURA3 promoter |
| pDB4544 | pNH605-pURA3-ScHFL1-I375A-GFP | pNH605 plasmid expressing ScHFL1-I375A-GFP from pURA3 promoter |
| pDB4545 | pNH605-pURA3-ScHFL1-W371AI375A-GFP | pNH605 plasmid expressing ScHFL1-W371AI375A-GFP from pURA3 promoter |
| pDB4546 | pNH605-pURA3-ScHFL1-D384A-GFP | pNH605 plasmid expressing ScHFL1-D384A-GFP from pURA3 promoter |
| pDB4547 | pNH605-pURA3-ScHFL1-Y387A-GFP | pNH605 plasmid expressing ScHFL1-Y387A-GFP from pURA3 promoter |
| pDB4548 | pNH605-pURA3-ScHFL1-W371AI375AD384AY387A-GFP | pNH605 plasmid expressing ScHFL1-W371AI375AD384AY387A-GFP from pURA3 promoter |
| pDB4549 | pNH605-pURA3-GFP | pNH605 plasmid expressing pURA3-GFP from pURA3 promoter |
| pDB4550 | pDUAL-P41nmt1-Hfl1-mCherry-SpAtg8 | pDUAL plasmid expressing Hfl1-mCherry-SpAtg8 from P41nmt1 promoter |
| pDB4551 | pDUAL-P41nmt1-Hfl1-Y398A-mCherry-SpAtg8 | pDUAL plasmid expressing Hfl1-Y398A-mCherry-SpAtg8 from P41nmt1 promoter |
| pDB4553 | pDUAL-P41nmt1-mCherry-SpAtg8 | pDUAL plasmid expressing mCherry-SpAtg8 from P41nmt1 promoter |
| pDB4554 | pDUAL-P41nmt1-Zhf1-mCherry | pDUAL plasmid expressing Zhf1-mCherry from  P41nmt1 promoter |
| pDB4555 | pDUAL-P41nmt1-Zhf1-mCherry-SpAtg8 | pDUAL plasmid expressing Zhf1-mCherry-SpAtg8 from  P41nmt1 promoter |
| pDB4556 | pDUAL-P41nmt1-Hmt1-mCherry | pDUAL plasmid expressing Hmt1-mCherry from  P41nmt1 promoter |
| pDB4557 | pDUAL-P41nmt1-Hmt1-mCherry-SpAtg8 | pDUAL plasmid expressing Hmt1-mCherry-SpAtg8 from  P41nmt1 promoter |
